# The Arabic medication-related burden quality of life (MRB-QoL) tool: Cross-cultural adaptation and content validation

**DOI:** 10.1016/j.rcsop.2024.100523

**Published:** 2024-10-10

**Authors:** Sundos Q. Al-Ebrahim, Jeff Harrison, Timothy F. Chen, Hamzah Alzubaidi, Mohammed A. Mohammed

**Affiliations:** a School of Pharmacy, Faculty of Medical and Health Sciences, The University of Auckland, Auckland, New Zealand; b Faculty of Pharmacy, The University of Sydney, Sydney, Australia; c College of Pharmacy, University of Sharjah, Sharjah, United Arab Emirates

**Keywords:** Medication-related burden, Quality of life, Translation, Cultural adaptation, Cross-cultural adaptation, Arabic, Delphi, Cognitive debriefing, Content validity

## Abstract

**Background:**

The Medication-Related Burden Quality of Life (MRB-QoL) is a 31-item valid and reliable patient-reported measure of medicine burden on functioning and well-being in people with long-term conditions (LTC).

**Objectives:**

To translate, culturally adapt, and content validate the MRB-QoL into Arabic.

**Methods:**

A rigorous approach to cross-cultural adaptation proposed by the International Society for Pharmacoeconomics and Outcomes Research (ISPOR) guideline was followed. After 3 forward translations and 2 backward translations, a multidisciplinary expert panel assessed the content validity (CV) of the items through a 2-round e-modified Delphi method followed by two-step cognitive debriefings with patients with LTC using think-aloud and probing techniques. An item-content validity index (I-CVI) score of ≥0.78 was considered acceptable. The original questionnaire developers and other researchers, as members of the review committee, reviewed and approved the Arabic version.

**Results:**

Five semantic and 3 cultural translation discrepancies were identified and resolved by rewording the items. The 2 backward translations did not reveal significant problems, and equivalence to the original tool was confirmed following committee review. The Arabic version showed acceptable CV parameters. *E*-modified Delphi involved 9 experts in round one and 7 in round 2. The I-CVI scores ranged from 0.67 to 1.0, and agreement was reached after 2 rounds. The CVI for the final version of the MRB-QoL was 0.96. Expert panel review showed that the MRB-QoL-Arabic version is relevant (CVI = 0.92), important (CVI = 0.97), clear (CVI = 0.98), and comprehensive in measuring the burden of medicines. Data from 5 cognitive interviews showed that items and concepts included in the Arabic version of the MRB-QoL are relevant to the targeted sample, clear, and easy to understand.

**Conclusion:**

The MRB-QoL Arabic version was developed and content validated. However, further evaluation of its other psychometric properties is necessary before it can be utilized in clinical and research settings. Using this tool will enable a more accurate understanding of the effects of treatment burden on patient well-being, thereby guiding care toward minimally disruptive medicine.

## 1 Introduction

The expansion of an aging population^1^, ^2^, ^3^ and the rise in the prevalence of long-term conditions (LTC)^2^^,^^4^^,^^5^ coupled with the proliferation of treatment guidelines that are focused on single disease state conditions rather than multimorbidity^1^^,^^2^^,^^6^ are common driving factors for the increase in trends of using multiple long-term medicines.^1^^,^^2^^,^^5^^,^^7^^,^^8^ Polypharmacy is commonly defined as the use of five or more medications regularly,^1^ and has now become a norm in LTC management.^9^^,^^10^ Polypharmacy may be clinically appropriate and necessary for many patients. However, polypharmacy is often burdensome to many patients especially the elderly and those on complex treatment regimens, and leads to prescribing cascades and poor health outcomes.^11^^,^^12^

Medication-related burden (MRB) is “a negative experience with medicine that can influence patients' health and well-being, beliefs and behaviors toward medicine and therapeutic care plans”.^13^ MRB can arise from medication routines, adverse events, the complexity of the regimen, and the impact of medicine on individuals' daily activities.^13^, ^14^, ^15^ Irrespective of the number and clinical appropriateness of the medicine, a patient may experience MRB, however, for many patients, the higher the number of medications adds the layers of complexity of MRB,^2^^,^^3^ which affects their functional and self-management capacity ultimately leading to poorer health-related quality of life (HRQoL) outcomes.^13^^,^^16^ Measuring the impact of MRB on well-being and quality of life can be considered one important tool for medicines optimisation to improve the health outcomes and quality of life of patients with LTC.

Objective measures are commonly used to assess medication burden^17^^,^^18^; however, being non-patient-centered, they are not suitable for capturing the views and perspectives of patients about how medication burden affects their health and well-being. The complete picture of the burden associated with long-term medicines can only be captured if objective measures are complemented by patient-reported measures of MRB.^13^^,^^16^^,^^19^

Over the past sixteen years, several patient-reported measures of MRB have been developed.^20^, ^21^, ^22^, ^23^, ^24^, ^25^, ^26^, ^27^ One such measure is the Medication Related Burden- Quality of Life (MRB-QoL). The MRB-QoL tool is a patient-reported measure of medicines' burden on functioning and well-being developed in Australia.^20^^,^^28^ The development of MRB-QoL was based on a holistic approach to understanding the impact of medications on individuals' lives, emphasizing not only on physical/physiological effects of medicines but also their impact on other key dimensions of well-being.^29^^,^^30^ The MRB-QoL has 31 items categorized into 5 domains: routine and regimen complexity (11 items), psychological burden (6 items), functional and role limitation (7 items), therapeutic relationship (3 items), and social burden (4 items).^20^^,^^30^ All items of the tool required respondents to express their level of agreement or disagreement with each statement on a 5-point Likert scale ranging from ‘1 = strongly agree,’ to ‘5 = strongly disagree’; higher scores indicated higher burden. Initial psychometric testing on community-dwelling adults in Australia showed that the MRB-QoL has good validity and internal consistency reliability.^20^

Implementing treatment burden measures in various countries has uncovered essential factors that affect the medication burden^31^, ^32^, ^33^, ^34^, ^35^ and has led to better adherence.^36^ Additionally, these measures have improved management strategies for patients with LTC.^37^, ^38^, ^39^ In the MENA region, LTC are a major public health concern.^40^ However, there is limited research on the treatment burden for patients with LTC in Arabic-speaking countries.^41^ A valid Arabic patient-centered measure recognizing and measuring MRB's impact is essential for optimising medication regimens and improving the well-being of patients with LTC. Currently, most Arabic patient-reported outcome measures (PROMs) are either disease-specific or generic, with few treatment-specific measures.^42^ Furthermore, only a few versions fully meet the standard criteria for CCA and psychometric testing, raising concerns about the quality of existing Arabic PROM.^42^ Three existing measures of treatment burden have been translated into Arabic, the Living with Medicines Questionnaire (LMQ),^43^ the Treatment Burden Questionnaire (TBQ),^44^^,^^45^ and the Multimorbidity Treatment Burden Questionnaire (MTBQ-A).^40^ The Arabic LMQ is lengthy, containing 41 questions, while the MTBQ-A is only suitable for patients with multiple LTCs, excluding those with a single condition. The Arabic TBQ lacks comprehensive CCA reporting and psychometric evaluation, while the English version^46^ requires high literacy levels due to complex wording. This highlights the need for more research to develop and adapt new treatment-specific measures for Arabic contexts. The MRB-QoL stands out for its versatility, being applicable to both multimorbid patients and those with a single LTC on long-term medication, thereby addressing a broader scope of MRB. Moreover, its strength and uniqueness lie in its development from 966 participant quotes across 34 qualitative studies, a comprehensive approach that is more robust than traditional methods relying on interviewing a single cohort of participants. There have been attempts to translate and culturally adapt the MRB-QoL measure in other languages (e.g. into German)^47^ but no work has been done on translating and culturally adapting the MRB-QoL tool for use in Arabic countries/people. This study addresses gaps in measuring MRB, issues with CCA quality, and the properties of existing Arabic PROMs. It also tackles the scarcity of treatment-specific Arabic PROMs and the limitations of current MRB measures by developing a new, comprehensive, medicine-specific quality-of-life measure for Arabic-speaking populations. This study aims to translate, culturally adapt, and content validate the MRB-QoL into Arabic.

## 2 Methods

### 2.1 Study design

**Fig. 1:**
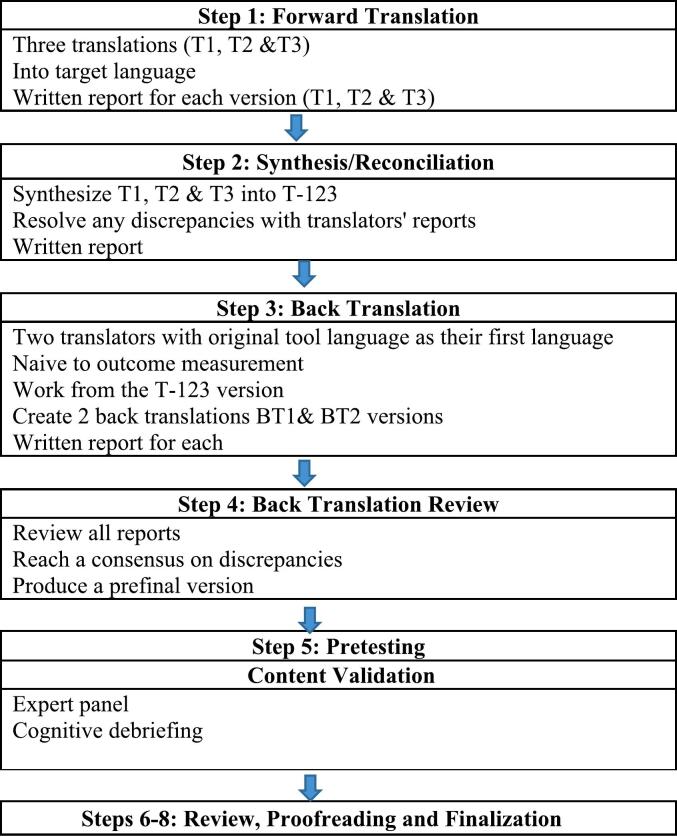
Development process of CCA.

This study employed two approaches, cross-cultural adaptation (CCA) and content validation. The research team followed the International Society for Pharmacoeconomics and Outcomes Research (ISPOR) guidelines for translation and cultural adaptation of patient-reported outcomes^48^ (Fig. 1). To ensure that all crucial aspects are taken into account throughout the design of the current study, we adhered to the COSMIN checklist for patient-reported outcome measures (PROMs).^49^ Two categories from this checklist were used: the general recommendations for designing a study on measurement properties, and the translation process (Table S1 supplementary data).

### 2.2 Translation and cultural adaptation of MRB-QoL

Two co-authors of this study (MAM and TFC)^20^ were developers of the original MRB-QoL tool and guided the development of the MRB-QoL Arabic version.

#### 2.2.1 Step 1: forward translation

Three bilingual translators independently translated the tool from the source language (i.e. English) into Arabic. All translators were native Arabic speakers, fluent in English, and had different backgrounds (2 experts in health research, and one registered medical translator). Translators were provided with instructions to (1) focus on conceptual/cultural equivalence, not just linguistic translation, (2) provide simple and clear translation, and (3) avoid terminology that might be ambiguous for end users to understand. Each translator provided a written report on the translation, including comments on complex phrases and unclear items, as well as explanations for their judgments.

#### 2.2.2 Step 2: synthesis

Translations from the 3 participants were merged and synthesized into a single translation. This process involved meeting with translators (2 out of 3) and a moderator (SA), and any discrepancies between translators were resolved by consensus to create a single reconciled version.

#### 2.2.3 Step 3: back translation

The reconciled MRB-QoL Arabic version was then back-translated into English by 2 bilingual and certified translators. Both translators independently translated the Arabic version into the original language (English) and had no prior information/knowledge about the original version of MRB-QoL. Both translators were native English speakers and fluent in Arabic.

#### 2.2.4 Step 4: reviewing back-translated version

The goal of CCA as a tool is to ensure cultural and linguistic equivalence between the original and the new version.^50^ The involvement of experts in the process plays a significant role in ensuring this.^50^, ^51^, ^52^ In this study, a review committee comprising the developers of the original MRB-QoL (MAM, TFC) and other co-authors (SQA, JH, HA) evaluated the back-translated version against the original English version. Discrepancies identified were discussed and resolved in consultation with translators. Following this, a preliminary Arabic version was produced.

#### 2.2.5 Step 5: pretesting

**Fig. 2:**
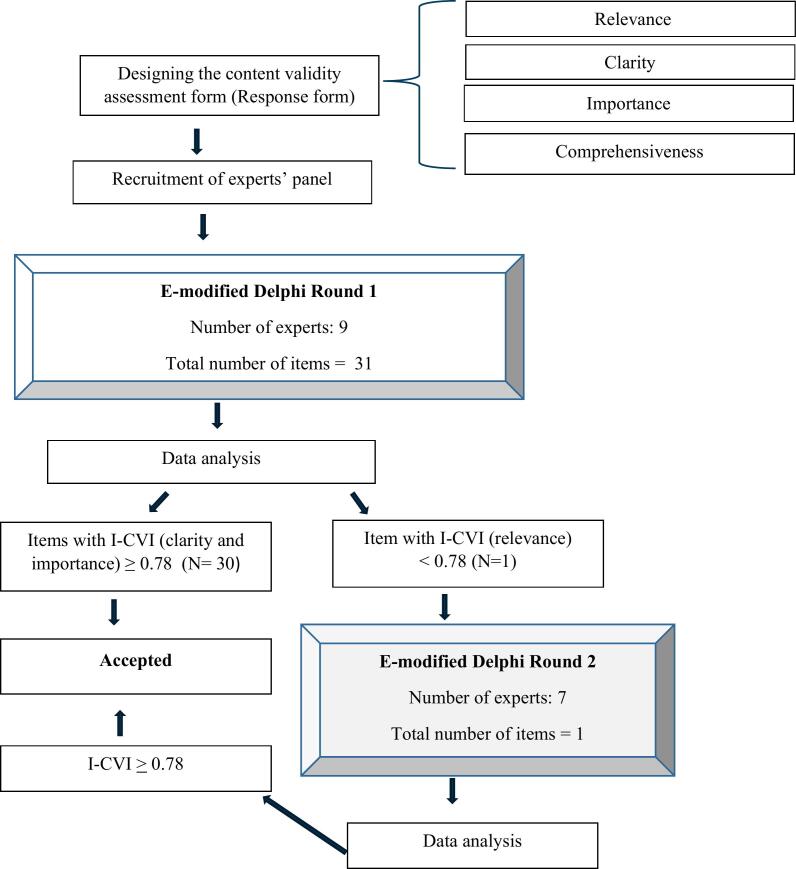
Flow diagram describing the process of the expert panel approach for content validity evaluation of the Arabic MRB-QoL.

The content validity (CV) testing of the MRB-QoL Arabic version was performed in 2 stages to ensure the Arabic version measures what it is supposed to measure.^53^ This involved an e-modified Delphi with an expert panel (Fig. 2) followed by cognitive debriefings with end users (i.e. Arabic-speaking individuals with LTC). Cognitive debriefing was conducted using a think-aloud approach and probing techniques.

Although reliability and construct validity are important, they were not included in this study. Our primary focus was on establishing CV due to its significant impact on other measurement properties.^54^ For example, irrelevant items can undermine internal consistency, structural validity, and interpretability, while missing concepts can weaken validity and responsiveness.^54^ High test-retest reliability and responsiveness do not guarantee the construct is accurately measured or that no crucial concepts are omitted.^54^ Following the confirmation of CV, detailed psychometric testing will be conducted in a future multistage study.

#### 2.2.6 Stage 1: e-Modified Delphi

Testing the CV was focused on assessing the following key areas: (1) the relevance^53^, ^54^, ^55^ of the MRB-QoL Arabic version in measuring the construct of interest, (2) the importance^53^ of the MRB-QoL in measuring the construct of interest, (3) clarity^53^^,^^55^ of the MRB-QoL i.e. whether the items are appropriately worded, and (4) comprehensiveness^53^^,^^54^ of the items in each domain and exploring new constructs not captured in the original version. The modified Delphi and e-Delphi methods were used. The e-Delphi technique is a consensus-building approach where feedback/responses from a panel of experts are gathered via online platforms.^56^ A modified Delphi^56^ method was used to build consensus on items of the MRB-QoL Arabic version. The e-modified Delphi approach was considered reasonable due to convenience as the expert panel was located in different countries and face-to-face interaction was not feasible. Even for local participants, face-to-face interaction was not a feasible option because of the COVID lockdown.

The Delphi study involved a steering committee and Delphi panelists. A steering committee comprised 5 researchers with expertise in the development and, evaluation of PROMs. The role of the committee was to provide advice on (1) Delphi protocol, (2) recruitment of the expert panel, (3) selection of checklist/questionnaire for the Delphi process, and (4) moderation and analysis of responses from Delphi rounds. The Delphi panel included clinicians with previous experience in using patient-reported measures in clinical settings and researchers who had at least one peer-reviewed publication related to CCA and/or content validation of an instrument.

Using purposive sampling, 14 panelists from 5 countries were invited via email. Arabic-speaking experts from the USA, Canada, Australia, UAE, and Jordan were recruited to obtain international perspectives regarding the content of the tool. Invited expert panel participants included clinical pharmacists, clinicians, academics, and researchers who fulfilled pre-specified inclusion criteria. The panelists received an invitation letter detailing the purpose of the e-modified Delphi, the process involved, and what is expected of them if they choose to participate, and a link to the consent form and a Qualtrics survey for content validation of the MRB-QoL.

The Qualtrics survey included the MRB-QoL domains and items (Table S2 supplementary data), and free text questions for general feedback on retaining or removing the items (comprehensiveness). The expert panel rated items in each domain on the following key areas using a 4-point Likert scale (1–4): (1) relevance, (2) importance, and (3) clarity of the items.

The readability and clarity of the survey were pilot-tested with 3 individuals who had prior experience in tool development. The feedback was used to modify the survey.

As the Delphi method involves an iteration of data collection and has a potential risk of participant attrition,^56^ an e-Delphi approach and limiting the number of rounds to 2 was used as a strategy to retain several participants. In addition, we used various techniques such as sending a reminder to participants to increase the response rate in both rounds.

The responses from expert panel members were entered into an Excel spreadsheet, and descriptive analysis was performed. Responses from open-ended questions were analyzed and modification (e.g. item rewording) was considered. The content validity index (CVI) was used for the analysis of the CV.^57^ An item-level content validity index (I-CVI) and the average content validity index (Ave-CVI) were calculated for relevance, importance, and clarity of the items. The proportion of agreement for comprehensiveness was calculated for each construct by dividing the number of experts who found no items needed to be added or removed by the total number of experts.^58^^,^^59^ For the second round Delphi, modified kappa k*^57^ was calculated alongside the CVI to adjust each I-CVI for chance agreement, providing a more accurate measure of interrater consensus in CV evaluation.^60^ We used the following equations^61^ to calculate k*, I-CVI, Ave-CVI, and Overall Ave-CVI:

1. k* = (I-CVI-p_c_)/(1- p_c_)

P_c_ is the probability of change agreement for each item and calculated as [N!/A! (N - A)!]X 0.5^N^, where N is the number of experts and A is the number of panelists who agree that the item is relevant.

2. I-CVI: Number of experts rating the item 3 or 4 / Total number of experts
3. Ave-CVI: Sum of individual I-CVIs / Total number of items
4. Overall Ave-CVI: Sum of all Ave-CVIs (relevance + importance + clarity) / 3

Survey findings were compiled and summarized using a recommended approach,^53^^,^^61^ where the cutoff ≥0.78 for I-CVI, ≥ 0.8 for Ave-CVI, and k* values ≥0.74 were considered excellent, indicating a high level of consistency between raters beyond what would be expected by chance alone. The I-CVI was used to determine whether an item should be included, rephrased, or removed in the first round.^61^ Items with an I-CVI of ≥0.78 for relevance, importance, and/or clarity, and no suggestions for rewording were retained. Items with a score of ≥0.78 on the I-CVI for relevance, importance, and/or clarity and identified by panelists as needing modification to improve clarity were rewarded. Items with an I-CVI < 0.78 for relevance, importance, and/or clarity were revised and considered for the second round. A second round of Delphi was focused on items requiring further modification to ensure the CV of the reviewed items.

#### 2.2.7 Stage 2: cognitive debriefing

Rigorous cognitive debriefing is essential for creating robust tools. Qualitative findings aid us and future researchers in detecting and understanding potential problems. Using both the think-aloud and probing methods ensures that patients consistently and accurately comprehend questions as intended.^62^ This technique enables a deeper exploration of patients' cognitive processes and facilitates the prompt resolution of misunderstandings.^63^ The purpose of cognitive debriefing was to ensure that the MRB-QoL Arabic version is culturally acceptable and understandable, for end users. A structured cognitive debriefing guide was developed in English, with expert input, and translated into Arabic. Debriefing was performed virtually via Zoom.

A purposive sampling approach was used to recruit individuals living with common LTC in the UAE. A minimum of 5 cognitive interviews are recommended for pretesting until saturation is attained,^48^^,^^64^, ^65^, ^66^ thus 5 participants were recruited in this study. Participants were considered for cognitive debriefing if they met the following inclusion criteria: 1) 18 years and older, 2) had at least one long-term condition, 3) were on treatment for their long-term condition, 4) able to provide written informed consent, and participate in a one-hour interview, and 5) fluent in Arabic.

Participants were invited to the study during their visit to the dental clinic in Sharjah, UAE. Participants interested in taking part in the study were sent participant information statements and consent forms via email. Upon obtaining consent, appointments were arranged with a member of the research team (SQA) for an interview via Zoom. A think-aloud technique and verbal probing were used to ensure participants understood the questions.^63^ The process started with participants completing the MRB-QoL questionnaire while thinking aloud and they were prompted to verbalize their thoughts as they responded to the question. For verbal probing, the interviewer asked how they interpreted specific words and why they chose a specific response category. Participants were also asked to provide feedback regarding the tool's ease of use, its length, and whether additional items needed to be included and any existing items needed to be removed.

#### 2.2.8 Steps 6–8: review, proofreading and finalization

Participant responses from Delphi and cognitive debriefing were summarized and reviewed by a steering committee before creating the final MRB-QoL Arabic version. Two experts in Arabic language and linguistics proofread the final version for typographic and grammatical errors.

### 2.3 Ethics approval

Ethics approval was obtained from the Auckland Health Research Ethics Committee at the University of Auckland, New Zealand (AH24337), the Research Ethics Committee of the UAE Ministry of Health and Prevention (MOHAP/DXB-REC/O.N·D/No.101/2022), and the Dubai Scientific Research Ethics Committee at the Dubai Health Authority, UAE (DSREC-01/2023_13).

## 3 Results

### 3.1 Translation and cultural adaptation

The MRB-QoL English version was sent to 3 forward translators without making any changes to the structure, format, or instructions of the tool. The team involved in forward translation identified clarity issues in some items. Some of these were due to the inherent differences in sentence structure and/or grammar between the English and Arabic languages, while others were attributed to semantic and cultural differences. To address these issues, the translators recommended incorporating more commonly used Arabic words and phrases. Specific examples of issues encountered during the translation and adaptation process are outlined in Table 1. For instance, a semantic issue was identified with the phrase “fitting medicine routines” (Item 4), which was initially translated as “managing.” This translation failed to capture the original concept of “fitting” (i.e., putting in or placing). The issue was resolved by selecting a more suitable and relevant term. Similarly, cultural discrepancies were also identified and resolved. The term “sexually frustrated” (Item 18) was problematic when translated literally, as it conveyed meanings that were culturally inappropriate and stigmatizing in Arabic. An equivalent, culturally appropriate Arabic term was used instead.

**Table 1:** Issues arise during the translation and adaptation process.

Translation issue	Item No.	Original English terms	Describe issue	How solved
Semantic	Instructions	“Consumer of health and medicine”	The literal translation of such an idiom provided misleading and uncommon expressions in Arabic.	The word “consumer of health and medicine” was used as an alternative explanation.
	Item 4	“Fitting medicine routines”	Fitting was translated and synthesized as “managing,” which has a different meaning/concept from the original term (Fitting = putting in or placing).	It was changed to a more suitable and relevant term.
	Item 7	“Manage medication regimen”	The literal translation of medication regimen does not provide the actual meaning of “A treatment plan that specifies the dosage, the schedule, and the duration of treatment”; instead, it reflects the meaning of “drug system.”	It was replaced with an alternative term with the conceptual meaning of medication regimen in Arabic.
	Item 9	“Convenient form”	The literal translation into Arabic does not preciously reflect the exact English meaning, so a further explanation of what the word “form” means was needed.	To clarify and better understand, we need to specify that the form is related to the pharmaceutical dosage form.
	Item 25	“Dignity”	The Literal translation was making the sentence structure weak.	Another alternative term was used.
Cultural	Item 18	“Sexually frustrated”	The literal translation of such a term provided misleading and uncommon expressions in Arabic. Which, when translated literally to Arabic, would refer mainly to sexual depression and would be culturally inappropriate and would be stigmatizing.	An equivalent Arabic term was used instead.
	Item 19	“Relax….sex”	The forward translation of Relax may indicate calmness and be not energetic in Arabic, which is entirely different from the original term (relax = less tense or anxious) and would be culturally inappropriate for sexual activity.	An equivalent appropriate Arabic term was used instead.
	Item 30	“Stigmatized”	The literal translation of such a term provided misleading expressions in Arabic.	An equivalent appropriate Arabic term was used instead.

After compiling results from the 3 forward translators, a reconciled version was generated, resolving any discrepancies among translators through discussion, and consensus. The back translation did not reveal significant deviation from the original English version in terms of conceptual equivalence. Following reviewing back translation, a prefinal Arabic version equivalent to the original version was created for field testing.

### 3.2 Content validation of the MRB-QoL- Arabic version

Table 2 provides the results of the I-CVI and decisions made to retain, remove, or modify an item based on expert rating. Of 14 experts invited to participate in the content validation, 9 participated (64 %) in the first round (Fig. 2). All 31 items met the predefined I-CVI threshold (≥ 0.78) for importance and clarity. However, one item (i.e. item 7) did not meet the minimum I-CVI threshold for relevance. The Delphi panel feedback on the item was reviewed by the research team and the item was reworded for the second-round expert panel rating. Seven of the 9 respondents from round one participated in the second round, resulting in a response rate of 77.8 %. All items rated in the second round met the predefined I-CVI threshold for relevance. In addition to the I-CVI results, expert panel feedback was reviewed before creating the revised MRB-QoL Arabic version. For comprehensiveness, the proportion of agreement was 1 for all constructs, as no deletion of items was suggested and no new items were added in the final MRB-QoL Arabic version.

**Table 2:** Content validity indices of items tested in the first and second rounds of the modified Delphi process.

**Item**	**Variables**	**Number of experts who endorsed item 3 or 4 (Total = 9)**	**I-CVI**	**Decision**	**Number of experts who endorsed item 3 or 4 (Total = 7)**	**I-CVI**	K*	Interpretation of K*	**I-CVI**	**Overall Ave-CVI**
		**Round 1**			**Round 2**				**Final version**	
1	Relevance	8	0.89	Accepted	_	_	_	_	0.89	0.96
	Importance	9	1.00	Accepted	_	_	_	_	1.00	
	Clarity	9	1.00	Accepted	_	_	_	_	1.00	
2	Relevance	9	1.00	Accepted	_	_	_	_	1.00	
	Importance	9	1.00	Accepted	_	_	_	_	1.00	
	Clarity	7	0.78	Accepted	_	_	_	_	0.78	
3	Relevance	7	0.78	Accepted	_	_	_	_	0.78	
	Importance	8	0.89	Accepted	_	_	_	_	0.89	
	Clarity	9	1.00	Accepted	_	_	_	_	1.00	
4	Relevance	7	0.78	Accepted	_	_	_	_	0.78	
	Importance	9	1.00	Accepted	_	_	_	_	1.00	
	Clarity	9	1.00	Accepted	_	_	_	_	1.00	
5	Relevance	8	0.89	Accepted	_	_	_	_	0.89	
	Importance	9	1.00	Accepted	_	_	_	_	1.00	
	Clarity	8	0.89	Accepted	_	_	_	_	0.89	
6	Relevance	8	0.89	Accepted	_	_	_	_	0.89	
	Importance	8	0.89	Accepted	_	_	_	_	0.89	
	Clarity	9	1.00	Accepted	_	_	_	_	1.00	
**7**	Relevance	6	0.67	Revised	7.00	1.00	1.00	Excellent	1.00	
	Importance	9	1.00	Accepted	_	_	_	_	1.00	
	Clarity	8	0.89	Accepted	_	_	_	_	0.89	
8	Relevance	7	0.78	Accepted	_	_	_	_	0.78	
	Importance	9	1.00	Accepted	_	_	_	_	1.00	
	Clarity	9	1.00	Accepted	_	_	_	_	1.00	
9	Relevance	8	0.89	Accepted	_	_	_	_	0.89	
	Importance	9	1.00	Accepted	_	_	_	_	1.00	
	Clarity	8	0.89	Accepted	_	_	_	_	0.89	
10	Relevance	7	0.78	Accepted	_	_	_	_	0.78	
	Importance	9	1.00	Accepted	_	_	_	_	1.00	
	Clarity	9	1.00	Accepted	_	_	_	_	1.00	
11	Relevance	9	1.00	Accepted	_	_	_	_	1.00	
	Importance	8	0.89	Accepted	_	_	_	_	0.89	
	Clarity	9	1.00	Accepted	_	_	_	_	1.00	
12	Relevance	9	1.00	Accepted	_	_	_	_	1.00	
	Importance	9	1.00	Accepted	_	_	_	_	1.00	
	Clarity	9	1.00	Accepted	_	_	_	_	1.00	
13	Relevance	9	1.00	Accepted	_	_	_	_	1.00	
	Importance	9	1.00	Accepted	_	_	_	_	1.00	
	Clarity	9	1.00	Accepted	_	_	_	_	1.00	
14	Relevance	9	1.00	Accepted	_	_	_	_	1.00	
	Importance	9	1.00	Accepted	_	_	_	_	1.00	
	Clarity	9	1.00	Accepted	_	_	_	_	1.00	
15	Relevance	9	1.00	Accepted	_	_	_	_	1.00	
	Importance	9	1.00	Accepted	_	_	_	_	1.00	
	Clarity	9	1.00	Accepted	_	_	_	_	1.00	
16	Relevance	9	1.00	Accepted	_	_	_	_	1.00	
	Importance	9	1.00	Accepted	_	_	_	_	1.00	
	Clarity	9	1.00	Accepted	_	_	_	_	1.00	
17	Relevance	8	0.89	Accepted	_	_	_	_	0.89	
	Importance	9	1.00	Accepted	_	_	_	_	1.00	
	Clarity	9	1.00	Accepted	_	_	_	_	1.00	
18	Relevance	8	0.89	Accepted	_	_	_	_	0.89	
	Importance	9	1.00	Accepted	_	_	_	_	1.00	
	Clarity	9	1.00	Accepted	_	_	_	_	1.00	
19	Relevance	8	0.89	Accepted	_	_	_	_	0.89	
	Importance	8	0.89	Accepted	_	_	_	_	0.89	
	Clarity	9	1.00	Accepted	_	_	_	_	1.00	
20	Relevance	9	1.00	Accepted	_	_	_	_	1.00	
	Importance	9	1.00	Accepted	_	_	_	_	1.00	
	Clarity	9	1.00	Accepted	_	_	_	_	1.00	
21	Relevance	9	1.00	Accepted	_	_	_	_	1.00	
	Importance	8	0.89	Accepted	_	_	_	_	0.89	
	Clarity	9	1.00	Accepted	_	_	_	_	1.00	
22	Relevance	9	1.00	Accepted	_	_	_	_	1.00	
	Importance	9	1.00	Accepted	_	_	_	_	1.00	
	Clarity	9	1.00	Accepted	_	_	_	_	1.00	
23	Relevance	9	1.00	Accepted	_	_	_	_	1.00	
	Importance	9	1.00	Accepted	_	_	_	_	1.00	
	Clarity	9	1.00	Accepted	_	_	_	_	1.00	
24	Relevance	9	1.00	Accepted	_	_	_	_	1.00	
	Importance	9	1.00	Accepted	_	_	_	_	1.00	
	Clarity	8	0.89	Accepted	_	_	_	_	0.89	
25	Relevance	8	0.89	Accepted	_	_	_	_	0.89	
	Importance	8	0.89	Accepted	_	_	_	_	0.89	
	Clarity	9	1.00	Accepted	_	_	_	_	1.00	
26	Relevance	8	0.89	Accepted	_	_	_	_	0.89	
	Importance	9	1.00	Accepted	_	_	_	_	1.00	
	Clarity	9	1.00	Accepted	_	_	_	_	1.00	
27	Relevance	8	0.89	Accepted	_	_	_	_	0.89	
	Importance	9	1.00	Accepted	_	_	_	_	1.00	
	Clarity	9	1.00	Accepted	_	_	_	_	1.00	
28	Relevance	8	0.89	Accepted	_	_	_	_	0.89	
	Importance	8	0.89	Accepted	_	_	_	_	0.89	
	Clarity	9	1.00	Accepted	_	_	_	_	1.00	
29	Relevance	9	1.00	Accepted	_	_	_	_	1.00	
	Importance	8	0.89	Accepted	_	_	_	_	0.89	
	Clarity	9	1.00	Accepted	_	_	_	_	1.00	
30	Relevance	9	1.00	Accepted	_	_	_	_	1.00	
	Importance	9	1.00	Accepted	_	_	_	_	1.00	
	Clarity	9	1.00	Accepted	_	_	_	_	1.00	
31	Relevance	9	1.00	Accepted	_	_	_	_	1.00	
	Importance	8	0.89	Accepted	_	_	_	_	0.89	
	Clarity	9	1.00	Accepted	_	_	_	_	1.00	
**Ave-CVI_ relevance**			**0.92**	**Ave-CVI_ relevance**					**0.98**	
**Ave-CVI_ importance**			**0.97**							
**Ave-CVI_ clarity**			**0.98**							

### 3.3 Cognitive debriefing

Following 2 rounds of e-modified Delphi, cognitive debriefing was conducted with 5 participants living with LTC (male = 3, female = 2). In terms of background, 3 out of 5 had bachelor's degrees, one completed secondary school, and another one completed primary school. Four were from Syria, Jordan, Egypt, and Libya residing in the UAE, while the fifth person was a UAE citizen. The mean (SD) age of the participants was 58 (10.61) years. The mean (SD) number of LTC and medications were 2.4 (1.02) and 5.6 (2.11) respectively. The diversity of the study participants enabled comprehensive evaluation of the adapted instrument by exploring the perspectives of end users of the tool across a range of demographics.

Participants considered the questionnaire easy to use with a reasonable completion time (on average, 10–15 min). Participants reported no significant problems with comprehension of the instrument except for one item where rewording the item was suggested to retain the intended meaning in the Arabic context. After reviewing, incorporating participant feedback, and proofreading, the final Arabic version of MRB-QoL was created with 31 items and 5 domains.

## 4 Discussion

This study is the first of CCA and content validation of the MRB-QoL measure for potential use in Arabic-speaking people/countries. International multiphase translation guidelines were followed in the CCA to ensure content accuracy, linguistic and cultural equivalence. Rigorous content validation assessment was implemented via the e-modified Delphi technique with an expert panel and cognitive debriefings with end users.

Arabic is the official language of 22 countries in the Middle East and North Africa (MENA) region. It is spoken as a native language by an estimated 420 million people worldwide (5 % of the world's population).^67^ The dialect and vocabulary of spoken Arabic vary from country to country; however, standard Arabic (spoken and written Fus-ha) is the same in all Arabic-speaking countries.^68^ As stated by AL-Ebrahim et al. (2023),^42^ out of the 201 translated and culturally adapted Arabic PROMs, 16 had multiple versions that were adapted in various Arabic-speaking countries using their respective spoken Arabic. To effectively utilize these cross-culturally adapted measures in different Arabic-speaking populations, it may be necessary to perform adaptations tailored to these PROMs to each culture. However, the MRB-Qol Arabic version can be used in all 22 Arab-speaking countries as its CCA is considered standard Arabic and includes diacritics (Tashkil). The inclusion of diacritics in the Arabic translation of the tools is essential as they provide readers with information about the nature of the word (verb, noun, adjective, etc.) and prevent potential mistranslations of certain words.^69^^,^^70^

Before 26 years ago, the utilization of HRQoL measures in research and clinical practice in the Arab world has been limited due to the lack of Arabic versions of the measures.^71^ It was only in 1998 that the first study on translation and validation of a generic HRQoL measure in Arabic was conducted.^72^ Since then, there has been a growing interest in CCA and validation of HRQoL measures into Arabs.^71^ Translation and adaption of a tool to a different culture is often complex and demands adequate resources. The process involves following a rigorous methodological approach for cultural adaptation to ensure translation quality and cultural and linguistic equivalence, and evaluation of psychometric properties of the culturally adapted measure.^50^ Ensuring that the original and a new version of CCA maintain cultural and linguistic equivalence requires a thorough translation process.^73^^,^^74^ Low-quality translation may have an impact on the transferability and applicability of the translated measures.^42^ The quality of CCA can be ensured by selecting appropriate translators, reconciling translators' feedback, piloting with end users, setting up an expert committee with expertise in the area, and involving original developers of a tool.^42^^,^^73^^,^^75^ The literature^42^ indicates that commonly used approaches in the CCA of PROMs into Arabic include the methods proposed by Guillemin et al. guidelines^76^ (17.8 %), Beaton guidelines^77^ (17.8 %), ISPOR guidelines^48^ (7 %), Brisling back translation model^78^ (6 %), and EORTC quality of life group translation procedure^79^ (3 %). In this study, we followed the ISPOR guidelines^48^ to ensure the methodological quality of CCA of the MRB-QoL measure.

The validation of an instrument is a process that encompasses various stages to undertake a compressive evaluation of psychometric properties.^80^ Content validity is often regarded as the most crucial measurement property, to ensure the extent to which the content of an instrument reflects the construct it is proposed to measure.^81^ This significance arises from the need for the items in an instrument to be relevant, clear, comprehensive, and important for the construct/area that an instrument proposed to measure. According to the FDA guidelines, it is advisable to evaluate PROMs CV before assessing other properties of the measurement.^82^ COSMIN guideline also suggests prioritizing consideration of content validation when comparing and evaluating the measurement properties of PROMs.^83^ In the present study, we followed the FDA and COSMIN recommendations by first assessing and ensuring the CV of MRB-QoL before evaluating its other psychometric properties. Thus, following this CCA and content validation of the MRB-QoL Arabic, future research should focus on the evaluation of other psychometric properties such as construct validity, criterion validity, internal consistency, test-retest reliability, measurement errors, sensitivity, and responsiveness, and its clinical utility in medicines optimisation services.

A rigorous evaluation process should be used to determine the CV of a newly developed instrument.^53^ Evidence on CV assures researchers that the assessment measures the intended constructs.^84^ Content validation of the MRB-QoL Arabic involved a multistage process, including quantitative evaluation by experts^66^ (9 in round 1, 7 in round 2) for relevance^53^, ^54^, ^55^, importance,^53^ clarity,^53^^,^^55^ and comprehensiveness^53^^,^^54^ and qualitative reviews by intended respondents.^66^ Involving both healthcare experts and patients in PROMs assessment ensures a thorough evaluation that takes into account clinical significance as well as patient viewpoints.^85^^,^^86^ This strategy improves the instrument's sensitivity, acceptability, and application in a variety of settings and demographics.^84^, ^85^, ^86^ Similarly, the MTBQ-A^40^ included quantified expert and qualitative patient evaluations of CV, but only 4 experts participated, and comprehensiveness was not assessed. The Arabic LMQ^87^ did not include quantified expert assessments and therefore does not meet the standards for CV testing, being more accurately referred to as having face validity.^58^ The Arabic TBQ lacks a comprehensive report of the entire CCA process and has not undergone psychometric evaluation.^44^^,^^45^

The culturally adapted and content-validated Arabic version of MRB-QoL retained the 31 items and 5 domains of the original tool. Our findings provide evidence that items of the MRB-QoL Arabic version adequately reflect the proposed constructs/domains. The average content validity index of the MRB-QoL total was 0.96 indicating that the translated Arabic version is important, relevant, and clear for measuring what is proposed to measure. Our results align with the findings for the MTBQ-A, which demonstrated a good CV with a CVI of 0.94.^40^ The findings of cognitive debriefing with 5 patients showed that the items of the MRB-QoL Arabic are clear and easy to understand. The overall sound level of comprehension of nearly all items in the measure stemmed from the original development of MRB-QoL.^20^ This development was grounded in the conceptualization of the area, which was informed by a meta-synthesis of MRB and patients' lived experiences with medicines,^13^ a meta-analysis of PC impact on HRQoL,^16^ and content analysis of HRQoL measures used in PC studies.^88^ While our study confirmed the CV robustness of the MRB-QoL Arabic version, Mendoza-Quispe et al. (2023)^89^ identified variability in CV quality among 7 original treatment burden instruments. Their findings indicated that CV was adequate for 4 instruments: MTBQ,^90^ Patient Experience with Treatment and Self-management (PETS),^91^ MRB-QoL,^20^ and LMQ.^92^ However, 3 instruments did not meet this standard. The Treatment Burden Questionnaire (TBQ),^46^^,^^93^ demonstrated insufficient CV because of inadequate clarity, whereas the Health Care Task Difficulty Questionnaire (HCTD)^94^ and Multimorbidity Illness Perceptions Scale (MULTIPLES)^95^ lacked comprehensiveness. The absence of a CV dimension can impact all other measurement properties.^54^ Irrelevant items can reduce internal consistency, structural validity, and the interpretability of the PROM. Missing concepts can diminish validity and responsiveness.^54^ Additionally, patients may become frustrated if they are asked irrelevant questions or if important questions are omitted, potentially leading to biased responses or low response rates.^96^^,^^97^

### 4.1 Implication for use in clinical practice and research

The Arabic MRB-QoL will be useful for clinicians, and researchers to optimise medication use in people with LTC and understand how MRBs may influence patients' health and well-being. The Arabic MRB-QoL is a substantial step toward measuring patient outcomes of clinical pharmacy services. Sound medication therapy decisions can only be made with good insights and attention to the lived experiences of patients with medicines. Generic and disease-specific HRQoL scales are nonsensitive to medications in polypharmacy patients.^98^ The Arabic MRB-QoL tool is a valuable contribution to research and practice that needs a treatment-specific outcome tool for measuring the burden of medicine on functioning and well-being. The Arabic MRB-QoL may be used in clinical practice as a screening, evaluation, and clinical decision-support tool to optimise LTC medication use in Arabic-speaking nations. This tool can be applied at various stages, such as initial patient evaluations, treatment, and follow-up, to enhance communication between patients and clinicians, prioritize patient-centered care, and improve service delivery. Routinely assessing patients with high MRB allows for targeted interventions to improve QoL and optimise medication management. Integrating an electronic version into electronic health records could further facilitate patient feedback on medication burden and well-being, streamline data collection through self-service devices during or between clinical visits, and support informed medication decisions. Healthcare providers require instruction to effectively administer the MRB-QoL tool. The instruction will provide comprehensive guidance on the administration and interpretation of the tool, which is inherently uncomplicated. In research, the Arabic MRB-QoL can be utilized to evaluate pharmaceutical care interventions and to identify key barriers and facilitators to medication optimisation in specific participant subgroups.

The development and content validation of the MRB-QoL Arabic version lay the foundation for future research to explore additional psychometric tests. These tests include exploratory and confirmatory factor analysis, various validity measures (known group, discriminant, convergent, criterion), and reliability measures (internal consistency, test-retest reliability, measurement error), as well as sensitivity and responsiveness. Future research should also determine clinically important cut-off points for MRB-QoL scores and evaluate their applicability across different populations. Lastly, the clinical utility of the MRB-QoL should be assessed in future trials.

### 4.2 Strengths and limitations

This is the first study to transculturally adapt and validate an Arabic version of the MRB-QoL tool. The principal strength of our study was the application of established guidelines for CCA and the involvement of the authors of the original version of the MRB-QoL tool (MAM, TFC) and a multidisciplinary research team. In addition, a rigorous method was also followed to explore the CV of the translated Arabic version, including an assessment of both cognitive debriefing and CVI.

Concerning the research methods, a limitation needs to be acknowledged. The interview-based cognitive debriefing data may have social desirability bias, where participants tend to answer in a socially acceptable manner.^99^ However, during the interviews, the researcher's role was limited to objectively reading the tools' items without giving a point of view, interfering, biasing, or leading the patient. Another limitation is the small sample size of 5 participants for cognitive debriefing, which may not capture all issues with the Arabic MRB-QoL tool, limiting the generalizability of the findings and potentially introducing selection bias. However, this sample size is regarded acceptable for qualitative study aiming at cognitive debriefing, because the primary purpose is to examine in-depth understanding and feedback rather than statistical representation. This sample size is consistent with qualitative research practices, which support using at least 5 participants,^48^^,^^64^, ^65^, ^66^ and was determined based on data saturation, where no new response difficulties emerged, ensuring sufficient data to assess the tool's content validity. Furthermore, the sample was diverse, representing several nationalities, which helps mitigate some of the limitations regarding generalizability and selection bias. A further limitation of this study is that only CV was assessed, while reliability and other types of validity were not evaluated. Consequently, the Arabic version of the MRB-QoL tool is not yet ready for use in clinical practice. Comprehensive psychometric testing, recommended for newly-adapted tools before their use in clinical practice and research, is necessary. However, detailed psychometric testing of the MRB-QoL Arabic version is planned by the research team.

## 5 Conclusion

The current study demonstrates that the developed Arabic version of the MRB-QoL is culturally appropriate for use among Arabic-speaking people with LTC. A panel of experts and cognitive debriefings with end users provided good evidence of a CV for the tool. The items and scales of the translated Arabic version of the MRB-QoL are relevant, clear, comprehensive, and essential to Arabic-speaking patients across various LTC and treatments. This study resulted in a measure suitable for further testing as part of a psychometric testing research project. Thus, future work is required to explore other types of psychometric properties. Integrating the MRB-QoL into clinical practice offers substantial benefits including improved patient-provider communication, unique insights into patient experiences, and support for providers in identifying and assisting patients with treatment burdens, thereby facilitating holistic medication optimisation.

## CRediT authorship contribution statement

**Sundos Q. Al-Ebrahim:** Writing – review & editing, Writing – original draft, Validation, Methodology, Data curation, Conceptualization. **Jeff Harrison:** Supervision. **Timothy F. Chen:** Validation, Supervision, Data curation, Conceptualization. **Hamzah Alzubaidi:** Supervision. **Mohammed A. Mohammed:** Writing – review & editing, Visualization, Validation, Supervision, Project administration, Methodology, Conceptualization.

## Declaration of competing interest

The authors declare the following financial interests/personal relationships which may be considered as potential competing interests:

Authors [MAM] and [TFC] are developers of the original MRB-QoL. Potential conflicts of interest may exist due to their role in its development. The remaining authors [SQA], [JH], and [HA] declare no conflict of interest. If there are other authors, they declare that they have no known competing financial interests or personal relationships that could have appeared to influence the work reported in this paper.

## Appendix A Supplementary data

Supplementary material Supplementary Table S1: COSMIN study design checklist for patient-reported outcome measurement instruments, including general recommendations for study design and translation process for the Arabic MRB-QoL tool. Supplementary Table S2: Content Validity Rubric and Assessment Response Form for evaluating item relevance, importance, and clarity in the Arabic MRB-QoL tool, alongside additional commentary fields to refine item comprehensiveness. Supplementary material
