# Supplementary material for: The Arabic medication-related burden quality of life (MRB-QoL) tool: Cross-cultural adaptation and content validation
Source: Explor Res Clin Soc Pharm. 2024 Oct 10;16:100523. doi: 10.1016/j.rcsop.2024.100523 (PMC11532770; doi:10.1016/j.rcsop.2024.100523)
Supplement: Supplementary material — Supplementary Table S1: COSMIN study design checklist for patient-reported outcome measurement instruments, including general recommendations for study design and translation process for the Arabic MRB-QoL tool. Supplementary Table S2: Content Validity Rubric and Assessment Response Form for evaluating item relevance, importance, and clarity in the Arabic MRB-QoL tool, alongside additional commentary fields to refine item comprehensiveness. [file mmc1.docx]

**Appendix A. Supplementary Data**

**Table S1**

COSMIN study design checklist for patient-reported Outcome Measurement Instruments.^1^COSMIN general recommendations for designing a study on measurement properties, and the translation process.


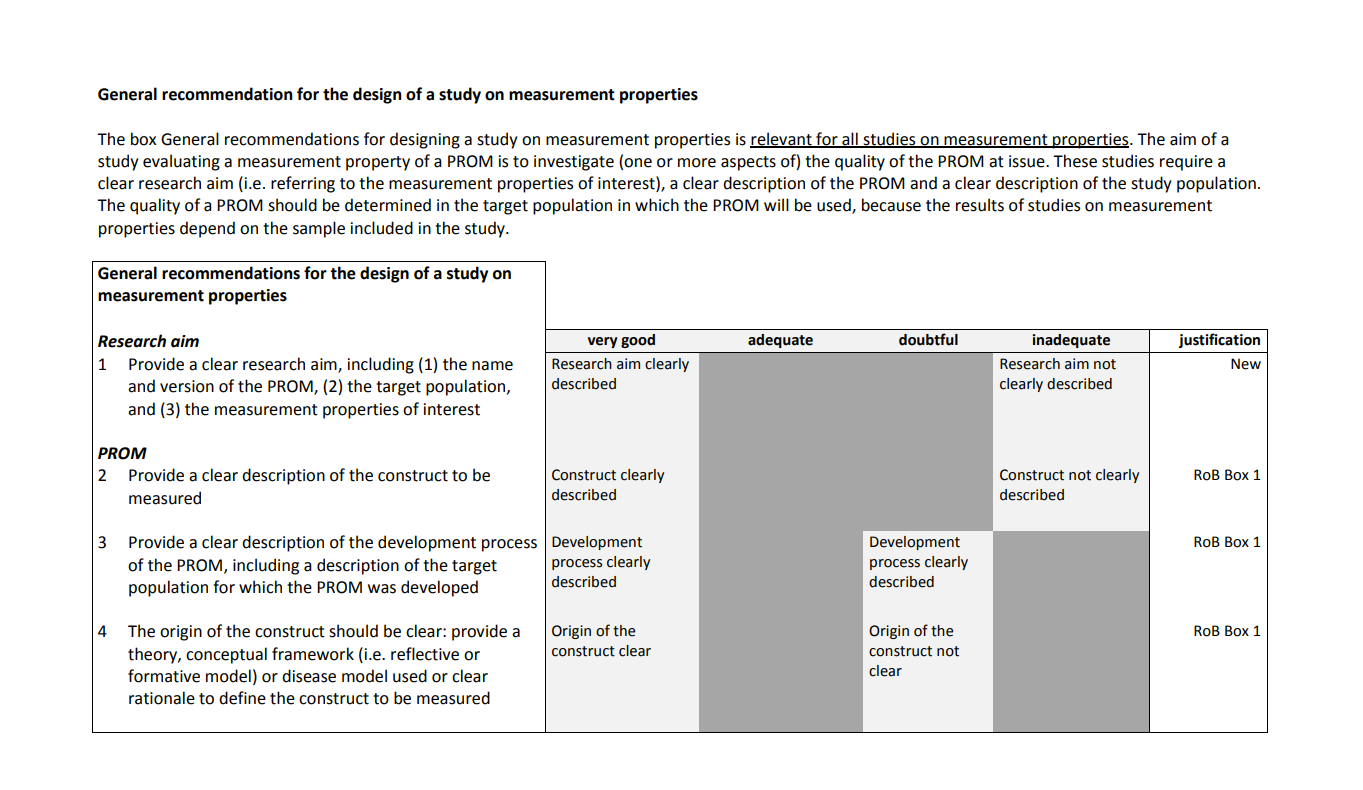


**
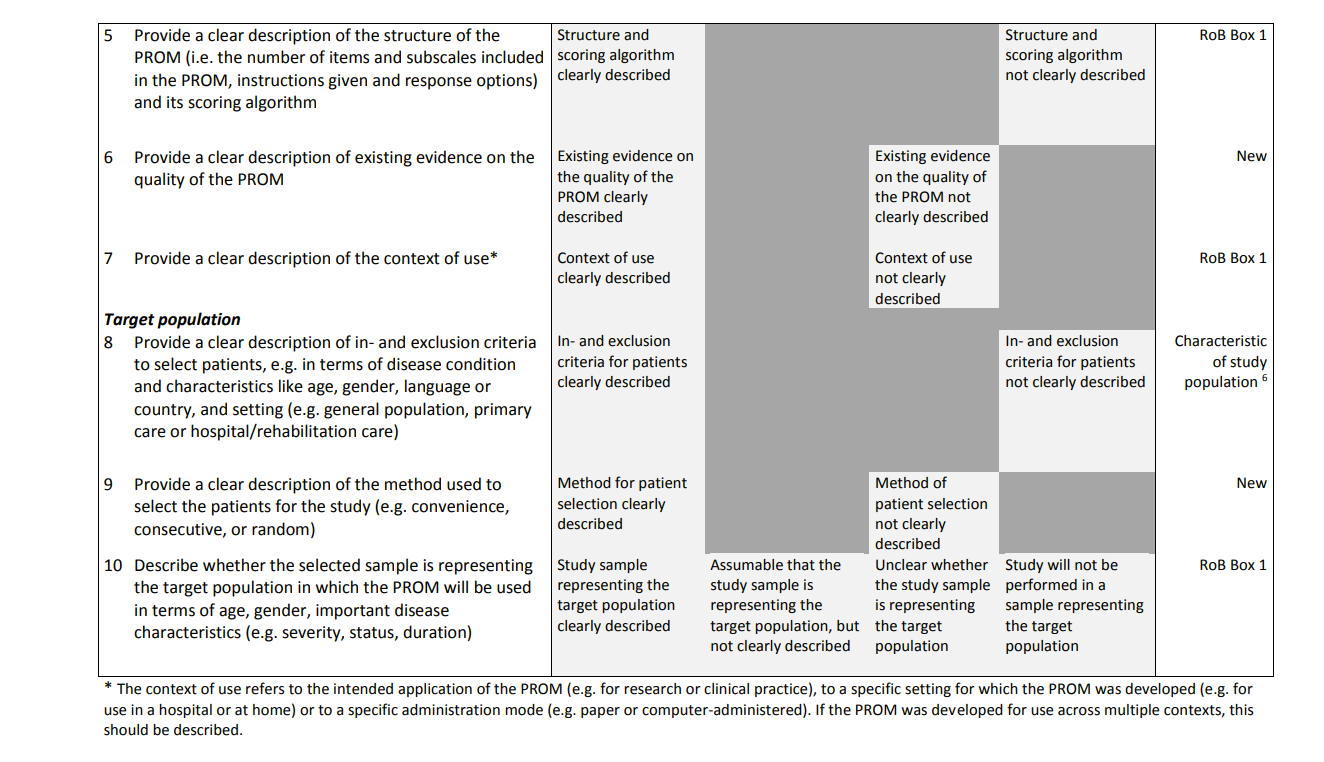
**


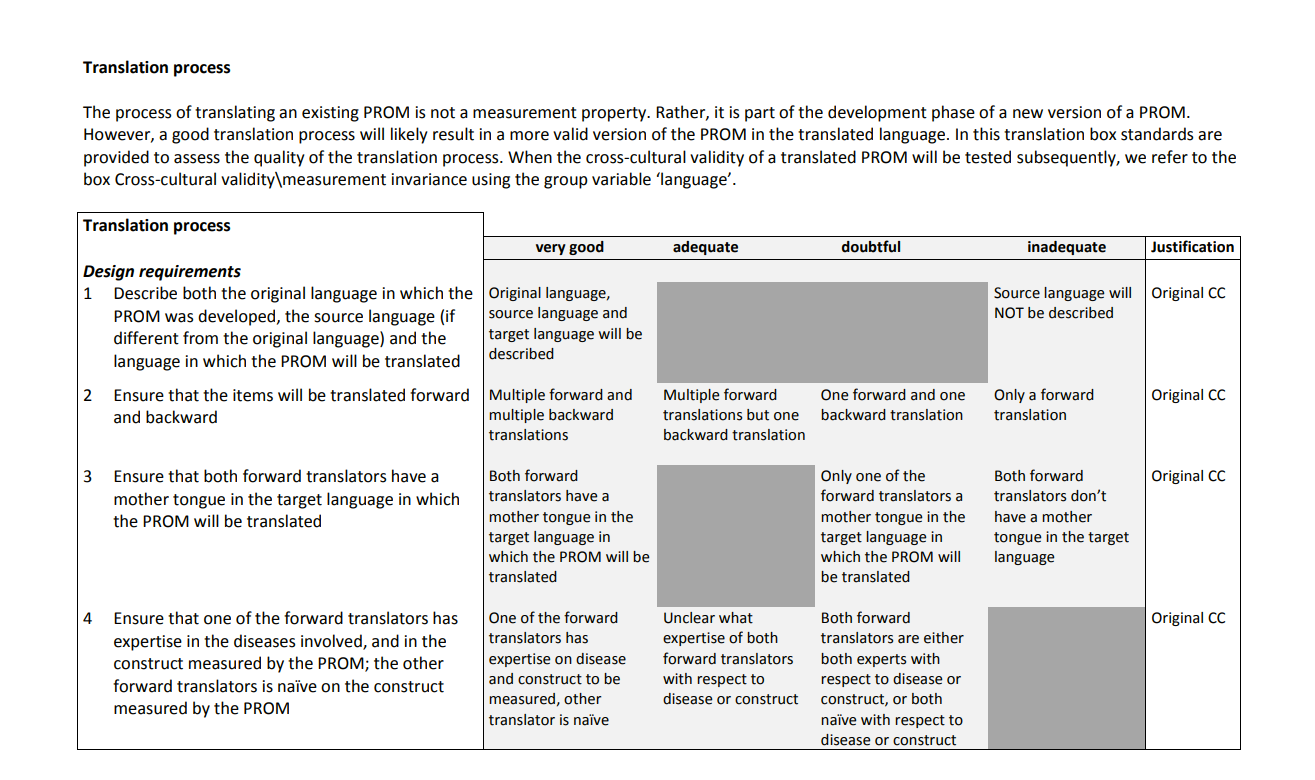


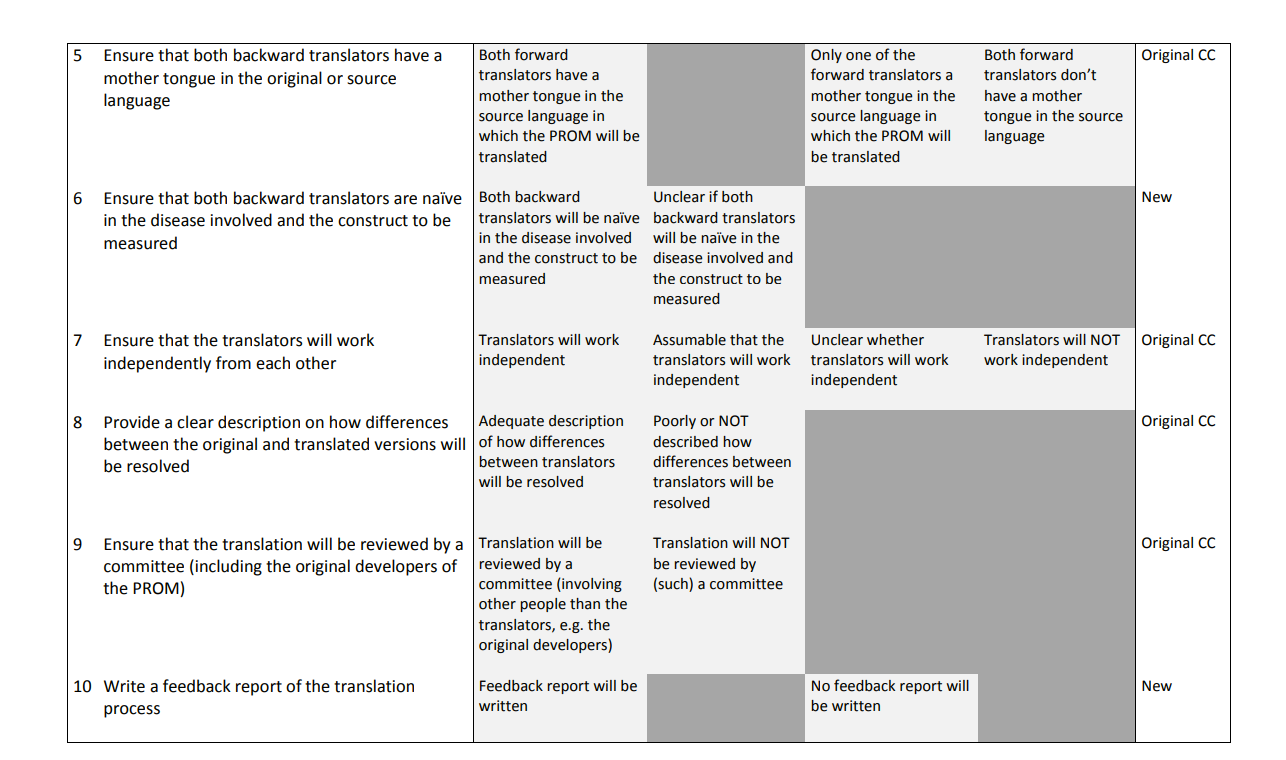


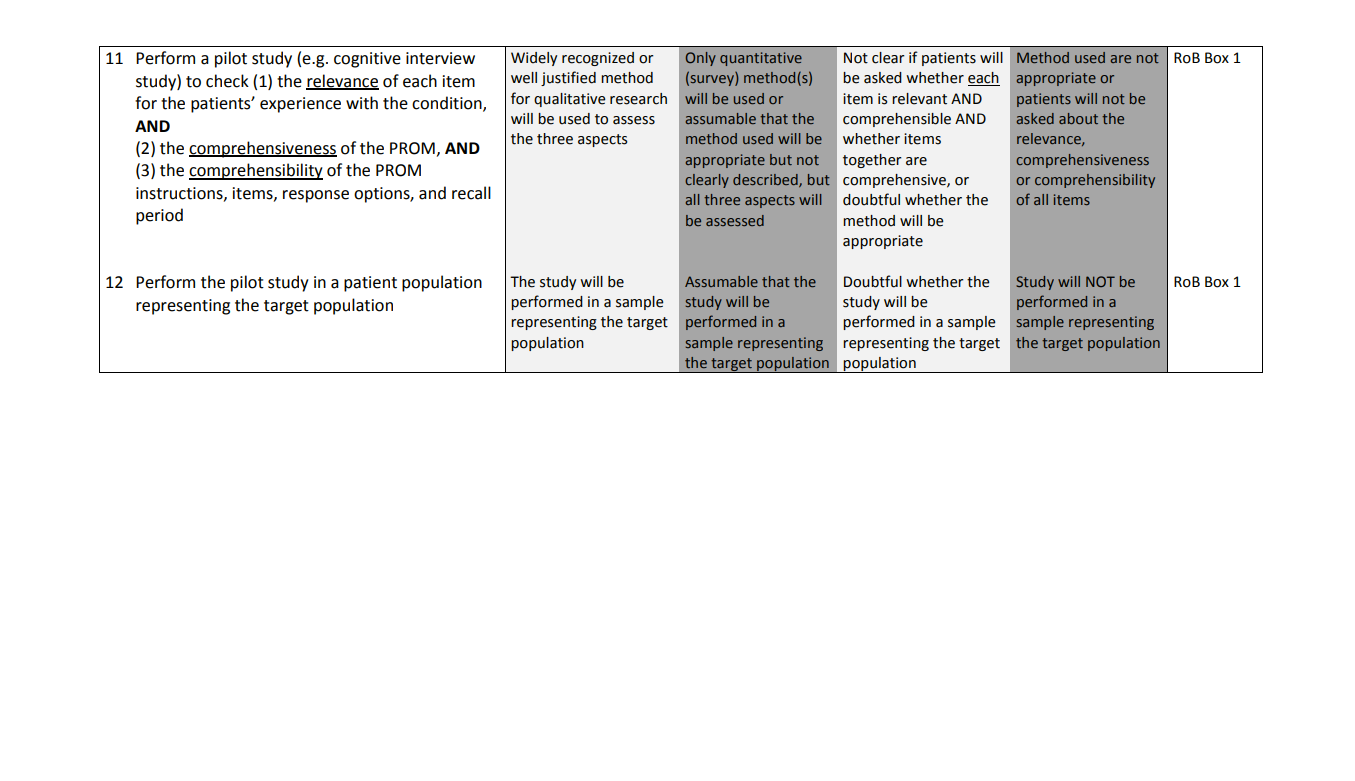


**Table S2**

Establishing Content Validity – Rubric/Assessment Response Form

Name of Reviewer: ___________________________________ Date: _________________________________________________________

INSTRUCTIONS: This measure is designed to evaluate the content validity of the Arabic version of MRB-Qol. Please rate each item as follows:

- Please rate the level of relevance of the item in measuring the aligned overarching construct on a scale of 1-4, with 4 being the most representative. Space is provided for you to comment on the item or suggest revisions.
- Please rate the importance of the item in measuring the aligned overarching construct on a scale of 1-4, with 4 being the most essential. Space is provided for you to comment on the item or suggest revisions.
- Please rate the level of clarity for each item on a scale of 1-4, with 4 being the clearest. Space is provided for you to comment on the item or suggest revisions.
- Finally, evaluate the comprehensiveness of the entire measure by indicating items that should be deleted or added. Thank you for your time.

| **Item No** | | **Overarching construct** | | **Item measuring the overarching construct** | | **Relevance of item in measuring the overarching construct**   - 1 = item is not relevant - 2 = item needs major revisions to be relevant - 3 = item needs minor revisions to be relevant - 4 = item is relevant | **Importance of items in measuring the overarching construct**   - 1 = item is not necessary to measure the construct - 2 = item is providing some information but is not essential to measure the construct - 3 = item is useful not but essential to measure the construct - 4 = item is essential to measure the construct | | **Clarity of item**   - 1 = item is not clear - 2 = The item needs major revisions to be clear - 3 = item needs minor revisions to be clear - 4 = item is clear | **Comments** | |
| --- | --- | --- | --- | --- | --- | --- | --- | --- | --- | --- | --- |
| **Construct 1: Routine and Regimen Complexity (RRC) (11 items (1-11))** | | | | | | | | | |  | |
| **Item-1** | **Organizing medicine routines (RRC-1)** | | Related Arabic item | | 1 2 3 4 | | | 1 2 3 4 | 1 2 3 4 |  |  |
| **Item-2** | **Keeping medicine records (RRC-2)** | | Related Arabic item | | 1 2 3 4 | | | 1 2 3 4 | 1 2 3 4 |  |  |
| **Item-3** | **Routine-managing (RRC-3)** | | Related Arabic item | | 1 2 3 4 | | | 1 2 3 4 | 1 2 3 4 |  |  |
| **Item-4** | **Fitting medicine routines (RRC-4)** | | Related Arabic item | | 1 2 3 4 | | | 1 2 3 4 | 1 2 3 4 |  |  |
| **Item-5** | **Interference with daily activities (RRC-5)** | | Related Arabic item | | 1 2 3 4 | | | 1 2 3 4 | 1 2 3 4 |  |  |
| **Item-6** | **Interference with daily activities (RRC-5)** | | Related Arabic item | | 1 2 3 4 | | | 1 2 3 4 | 1 2 3 4 |  |  |
| **Item-7** | **Simplicity of medicine regimen (RRC-7)** | | Related Arabic item | | 1 2 3 4 | | | 1 2 3 4 | 1 2 3 4 |  |  |
| **Item-8** | **Medicine-instructions (RRC-8)** | | Related Arabic item | | 1 2 3 4 | | | 1 2 3 4 | 1 2 3 4 |  |  |
| **Item-9** | **Regimen-convenience (RRC-9)** | | Related Arabic item | | 1 2 3 4 | | | 1 2 3 4 | 1 2 3 4 |  |  |
| **Item-10** | **Medicine and daily life schedules (RRC-10)** | | Related Arabic item | | 1 2 3 4 | | | 1 2 3 4 | 1 2 3 4 |  |  |
| **Item-11** | **Medicine-package (RRC-11)** | | Related Arabic item | | 1 2 3 4 | | | 1 2 3 4 | 1 2 3 4 |  |  |
| What additional items would you recommend including to measure the construct? If you have no suggestions, please enter “none.” | | | | | | | | | |  |  |
| What additional items would you recommend deleting? If you have no suggestions, please enter “none.” | | | | | | | | | |  |  |
| Please provide any additional information you believe may be useful in assessing the identified construct with this instrument. If you have no suggestions, please enter “none.” | | | | | | | | | |  |  |
| **Construct 2: Psychological Burden (PsyB) ( 6 Items (12-17))** | | | | | | | | | |  |  |
| **Item-12** | **Long term-medicine (PsyB-1)** | | Related Arabic item | | 1 2 3 4 | | | 1 2 3 4 | 1 2 3 4 |  |  |
| **Item-13** | **Number of medicines (PsyB-2)** | | Related Arabic item | | 1 2 3 4 | | | 1 2 3 4 | 1 2 3 4 |  |  |
| **Item-14** | **Long term- impact (PsyB-3)** | | Related Arabic item | | 1 2 3 4 | | | 1 2 3 4 | 1 2 3 4 |  |  |
| **Item-15** | **Medicine reminds health conditions (PsyB-4)** | | Related Arabic item | | 1 2 3 4 | | | 1 2 3 4 | 1 2 3 4 |  |  |
| **Item-16** | **Medicine-interactions (PsyB-5)** | | Related Arabic item | | 1 2 3 4 | | | 1 2 3 4 | 1 2 3 4 |  |  |
| **Item-17** | **Medicine-signifies problem (PsyB-6)** | | Related Arabic item | | 1 2 3 4 | | | 1 2 3 4 | 1 2 3 4 |  |  |
| What additional items would you recommend including to measure the construct? If you have no suggestions, please enter “none.” | | | | | | | | | |  |  |
| What additional items would you recommend deleting? If you have no suggestions, please enter “none.” | | | | | | | | | |  |  |
| Please provide any additional information you believe may be useful in assessing the identified construct with this instrument. If you have no suggestions, please enter “none.” | | | | | | | | | |  |  |
| **Construct 3: Functional and Role Limitation (FRL) ) (7 Items (18-24))** | | | | | | | | | |  |  |
| **Item-18** | **Sexual relationship (FRL-1)** | | Related Arabic item | | 1 2 3 4 | | | 1 2 3 4 | 1 2 3 4 |  |  |
| **Item-19** | **Sexual activity (FRL-2)** | | Related Arabic item | | 1 2 3 4 | | | 1 2 3 4 | 1 2 3 4 |  |  |
| **Item-20** | **Medicine and physical health (FRL-3)** | | Related Arabic item | | 1 2 3 4 | | | 1 2 3 4 | 1 2 3 4 |  |  |
| **Item-21** | **Medicine and night sleep (FRL-4)** | | Related Arabic item | | 1 2 3 4 | | | 1 2 3 4 | 1 2 3 4 |  |  |
| **Item-22** | **Medicine and physical activities (FRL-5)** | | Related Arabic item | | 1 2 3 4 | | | 1 2 3 4 | 1 2 3 4 |  |  |
| **Item-23** | **Medicine- impact on work (FRL-6)** | | Related Arabic item | | 1 2 3 4 | | | 1 2 3 4 | 1 2 3 4 |  |  |
| **Item-24** | **Comfort and side effect (FRL-7)** | | Related Arabic item | | 1 2 3 4 | | | 1 2 3 4 | 1 2 3 4 |  |  |
| What additional items would you recommend including to measure the construct? If you have no suggestions, please enter “none.” | | | | | | | | | |  |  |
| What additional items would you recommend deleting? If you have no suggestions, please enter “none.” | | | | | | | | | |  |  |
| Please provide any additional information you believe may be useful in assessing the identified construct with this instrument. If you have no suggestions, please enter “none.” | | | | | | | | | |  |  |
| **Construct 4: Therapeutic Relationship (3 Items (25-27))** | | | | | | | | | |  |  |
| **Item-25** | **Respect and dignity (TR-1)** | | Related Arabic item | | 1 2 3 4 | | | 1 2 3 4 | 1 2 3 4 |  |  |
| **Item-26** | **Decisions and considerations (TR-2)** | | Related Arabic item | | 1 2 3 4 | | | 1 2 3 4 | 1 2 3 4 |  |  |
| **Item-27** | **Decisions and engagement (TR-3)** | | Related Arabic item | | 1 2 3 4 | | | 1 2 3 4 | 1 2 3 4 |  |  |
| What additional items would you recommend including to measure the construct? If you have no suggestions, please enter “none.” | | | | | | | | | |  |  |
| What additional items would you recommend deleting? If you have no suggestions, please enter “none.” | | | | | | | | | |  |  |
| Please provide any additional information you believe may be useful in assessing the identified construct with this instrument. If you have no suggestions, please enter “none.” | | | | | | | | | |  |  |
| **Construct 5: Social Burden (SB) (4 Items (28-31))** | | | | | | | | | |  |  |
| **Item-28** | **Lived experience with others (SB-1)** | | Related Arabic item | | 1 2 3 4 | | | 1 2 3 4 | 1 2 3 4 |  |  |
| **Item-29** | **Public perception (SB-2)** | | Related Arabic item | | 1 2 3 4 | | | 1 2 3 4 | 1 2 3 4 |  |  |
| **Item-30** | **People and stigma (SB-3)** | | Related Arabic item | | 1 2 3 4 | | | 1 2 3 4 | 1 2 3 4 |  |  |
| **Item-31** | **Self-stigma (SB-4)** | | Related Arabic item | | 1 2 3 4 | | | 1 2 3 4 | 1 2 3 4 |  |  |
| What additional items would you recommend including to measure the construct? If you have no suggestions, please enter “none.” | | | | | | | | | |  |  |
| What additional items would you recommend deleting? If you have no suggestions, please enter “none.” | | | | | | | | | |  |  |
| Please provide any additional information you believe may be useful in assessing the identified construct with this instrument. If you have no suggestions, please enter “none.” | | | | | | | | | |  |  |

**Thank you for your time**

References

**1.** Mokkink LB, Prinsen C, Patrick DL, et al. COSMIN Study Design checklist for Patient-reported outcome measurement instruments. *Amsterdam, The Netherlands.* 2019:1-32.
